# Muscular tension as an indicator of acute stress in horses

**DOI:** 10.14814/phy2.15220

**Published:** 2022-03-21

**Authors:** Ellen M. Rankins, Helio C. Manso Filho, Karyn Malinowski, Kenneth H. McKeever

**Affiliations:** ^1^ Equine Science Center Department of Animal Science Rutgers The State University of New Jersey New Brunswick New Jersey USA; ^2^ Departamento de Zootecnia Universidade Federal Rural de Pernambuco (UFRPE) Recife‐PE Brasil

**Keywords:** acute stress, behavior, equine, surface EMG

## Abstract

Horses’ muscular tension during acute stress remains unexplored. Our aim was to assess muscular, behavioral, cortisol, and hematocrit responses to social isolation (ISO), novel object exposure (NOV), and sham clipping (CLIP). Altered stress responses were expected. Eight mature Standardbred horses (four mares and four geldings) were exposed to acute stressors and a control period (CON) in a balanced, replicated 4×4 Latin Square experimental design with 3 min treatment periods and 10 min washout periods. Surface electromyography collected from the *masseter*, *brachiocephalas*, *cervical trapezius*, and *longissimus dorsi* was processed to derive average rectified value (ARV) and median frequency (MF) during the initial, middle, and final 30 s of treatments. ARV and MF data were log transformed then analyzed using a mixed model, repeated measures ANOVA along with plasma cortisol and hematocrit. Behavior data were analyzed using a negative binomial distribution mixed model ANOVA. CLIP resulted in greater (*p* < 0.05) log ARV in the *masseter* (1.5 + 1.5%, mean + SD) and *brachiocepahlas* (2.2 + 2.0%) than CON (−1.2 + 1.4%, 0.1 + 1.5%). ISO resulted in greater (*p* < 0.05) log ARV in the *masseter* (0.2 + 1.3%) and *cervical trapezius* (0.6 + 1.3%) than CON (−1.2 + 1.4%, −1.0 + 1.7%). ISO increased (*p* < 0.05) the total number of stress‐related behaviors and hematocrit. No changes in cortisol were observed. We suggest that muscular tension can be used as an indicator of acute stress in horses. Incorporating muscle activity into an array of measurements may provide a more nuanced understanding of stress responses.

## Graphical abstract

We examined horses’ muscular, behavioral, endocrine, and hematological responses to three acute stressors. Stressors elicited differential responses. Muscular activity did increase in response to some acute stressors indicating muscular activity measured via surface EMG in the horse may be used as an indicator of acute stress. We recommend using this novel approach in tandem with other measures to provide a more nuanced understanding of stress in horses.

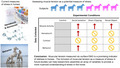

## 1 INTRODUCTION

All species experience stress – a disruption in the body's homeostasis provoked by mental, emotional, or physical strain resulting in physiological and behavioral responses to the stimuli. We often think of stress as something needing to be eliminated or minimized. In reality, stress can be positive, eustress, or negative, distress. Distress, stress that is damaging or unpleasant, is often what people think of when the word stress is used.

Stress, whether eustress or distress, is inextricably linked to welfare and its assessment in horses and other animals. Distress, particularly chronic distress, makes the achievement of a positive state of welfare impossible (Veissier & Boissy, 2007). Maintaining and improving horse welfare is critical to the long‐term sustainability of the equine industry as well as attaining peak horse performance (Bartolomé et al., 2013; Lesimple, 2020). To meet these goals, we need to understand the effects of management and training practices on horses’ stress responses and the role acute and chronic stressors play in determining a horse's state of welfare.

Stress in horses can be evaluated via behavioral or physiological measures (König von Borstel et al., 2017). Behavioral responses to stress often exhibit greater plasticity and range than physiological responses (König von Borstel et al., 2017). Physiological measures typically reflect activation of either the hypothalamic–pituitary–adrenal (HPA) axis or the sympathetic–adrenal–medulla (SAM) axis (König von Borstel et al., 2017). Activation of the HPA axis results in increased plasma and salivary cortisol (König von Borstel et al., 2017). The primary products of the SAM axis are the catecholamines, norepinephrine and epinephrine, which cause further physiological changes in the body such as increased heart rate and alterations in heart rate variability (König von Borstel et al., 2017; Myrtek, 2004). In horses, sympathetic activation also results in an increase in hematocrit through the actions of the catecholamines (McGowan & Hodgson, 2014). A pool of red blood cells is stored in the spleen. When sympathetic activation occurs, the spleen contracts, thereby forcing more red blood cells into circulation and increasing the hematocrit (McGowan & Hodgson, 2014). More recent work has suggested the use of other physiological measures such as spontaneous eye blink rate and eye temperature to assess stress in the horse (McGreevy et al., 2012; Mott et al., 2020; Yarnell et al., 2013). The rationale for exploring other measures of stress is two‐fold. First, measuring more than one variable in assessing stress is useful as it allows for a more nuanced understanding of the horse's stress response including whether a stressor results in eustress or distress for an animal and may assist in the interpretation of responses exhibiting plasticity, such as behavioral responses (König von Borstel et al., 2017). Additionally, the development and validation of non‐invasive measures can alleviate concerns of the measures themselves inducing stress in the horse.

A non‐invasive measure of stress which remains unexplored in the horse is muscle tension. Human studies associate muscular tension, especially in the jaw, neck, and shoulder, with mental stress and psychological disorders (Davidowitz et al., 1955; Lundberg et al., 1994; Pétursson, 1962; Sainsbury & Gibson, 1954). Resting muscle activity or tension along the spine has been explored as an indicator of welfare in horses, but the study did not examine the effects of stress on muscle tension (Lesimple et al., 2012). The measurement of muscle activity along the spine in this study makes further investigation of muscle activity in this location attractive (Lesimple et al., 2012). Facial muscles also present an attractive target in which to study muscle tension – think of clenching your jaw (Pétursson, 1962; Sainsbury & Gibson, 1954). The face and its multitude of muscles make this feature one of the most expressive in the body and the horse is no exception. Pain can be assessed and scored based on facial expression alone (Gleerup et al., 2015).

To explore novel measures of stress in the horse, it must be exposed to conditions known to elicit a stress response and cause distress, at least temporarily to the horse. Many different stress eliciting conditions can be found in the literature, including sham clipping, social isolation, and novel object exposure. Sham clipping, applying electric clippers to the horse's body, has been used in other recent validations of measures of stress in the horse (Mott et al., 2020; Yarnell et al., 2013). Social isolation has a long history of use in assessing stress and is particularly relevant to the horse as it is a gregarious species (Mal et al., 1991). Exposure to a novel object elicits a fear response in the horse, although the particular response elicited may be dependent on the object presented (Bulens et al., 2015; Lansade et al., 2008b).

The aim of this study was to determine the effects of acute stress in the form of social isolation, novel object exposure, and sham clipping on muscle activity in facial, neck, shoulder, and back muscles, behavior, plasma cortisol, and hematocrit in the horse. We hypothesized that acute stress would alter muscle activity, behavior, plasma cortisol, and hematocrit in horses as compared to a standing control.

## 2 MATERIALS AND METHODS

### 2.1 Animals

Eight mature Standardbred horses [four mares and four geldings, 13 years (SD 5.7), 495 kg (SD 42)] were used in the study. Horses were housed by sex in 0.8 ha dry lots at the Rutgers University Large Animal Farm in New Brunswick, New Jersey. Horses were fed a commercial maintenance ration (maximum 1% body weight) and had *ad libitum* access to an alfalfa and grass hay mixture. Horses had continual access to water and trace mineralized salt blocks. Prior to the start of the study, horses were familiarized with the study procedures and handling by the study staff. Experimental procedures were approved by the Rutgers University Institutional Animal Care and Use Committee.

### 2.2 Experimental design

To assess the behavioral, hormonal, and muscular responses of horses to stressful situations, a replicated 4×4 balanced Latin Square experimental design was used. Horses were exposed to four conditions – control (CON), novel object (NOV), sham clipping (CLIP), and social isolation (ISO) – in 3‐min periods with 10‐min washout periods between each treatment period. Each horse was exposed to all four conditions on one day with testing occurring over 4 days. Testing occurred between 1100 and 1400. The order in which horses were tested was randomized. In the CON condition, horses remained loose in a box stall (3.7 × 3.7 m) with companion horses in the adjoining stalls (visual, auditory, and tactile contact). During the NOV condition, horses were fitted with a halter and lead line. A handler led the horse from the box stall and onto a novel object (blue tarp secured to the floor). The lead line was loosely held for the 3‐min period and the horse redirected if it attempted to exit the tarp. The horse remained within close proximity (visual and auditory contact) with the companion horses. The same handler was used for all horses and conditions. In the CLIP condition, horses were fitted with a halter and lead line and led from the stall by the handler. Electrical clippers were turned on and applied to the horse's upper neck (slightly caudal to the base of the ears). The side on which the clippers were applied (left and right) was periodically switched during the 3‐min period. Pressure was applied to the lead line if the horse attempted to move away from the clippers. Horses remained in close proximity (visual and auditory contact) to the companion horses. During ISO, horses remained loose in the box stall. Companion horses were removed from the barn. At the end of the period, companion horses were returned to the barn. Access to feed and water was removed during the testing period. Companion horses were provided with *ad libitum* access to fresh water and hay in their stalls. Horses were returned to their respective dry lots following completion of the last blood collection and removal of data collection equipment.

### 2.3 sEMG data collection

Prior to the start of the study, the hair at the sEMG attachment sites was clipped to a length of 0.6 mm (Licka et al., 2009; Zsoldos et al., 2010). sEMG attachment sites were scrubbed with a 70% alcohol solution and allowed to air dry. Self‐adhesive sEMG electrodes (Ag/AgCl, 1.3‐cm diameter, 2‐cm inter‐electrode distance, Noraxon, Scottsdale, AZ, USA) and telemetric transmitter units (Ultium EMG, Noraxon, Scottsdale, AZ, USA) were attached to the right and left *masseter*, *brachiocephalas*, *cervical trapezius*, and *longissimus dorsi* using the supplied self‐adhesive backing. The electrode for the *masseter* was placed approximately 5 cm cranial to the caudal edge of the mandible (Williams et al., 2014). The electrode for the *brachiocephalas* was placed in the center of the muscle belly parallel to the muscle fibers as determined by manual palpation of surrounding landmarks and approximately 16 cm cranial to the posterior part of the greater tubercle of the humerus (point of the shoulder) (Kienapfel, 2015; Robert et al., 1998). The electrode for the *cervical trapezius* was placed parallel to the muscle fibers approximately 6 cm cranial to the proximal end of the spine of the scapula (Robert et al., 1998). The electrode for the *longissimus dorsi* was placed approximately 5 cm lateral to the spinous process of T12 (Robert et al., 1998, 2010). sEMG transmitter units were further secured with electrical tape (3 M, Saint Paul, MN, USA) and self‐adhesive bandages (VetRap™, 3 M, Saint Paul, MN, USA) to prevent detachment during locomotion or other activities. sEMG data were collected continuously during each of the four treatment periods. Telemetric sEMG units were connected wirelessly to a laptop. Data were viewed and collected in real time at a sampling frequency of 2000 Hz (MR 3.14™, Noraxon, Scottsdale, AZ, USA) with application in an analog gain of 500 for amplification. Prior to data collection, impedance was measured using the software system (MR 3.14™, Noraxon, Scottsdale, AZ, USA). Data collection only proceeded if impedance was less than 10kΩ. As data were collected, a high‐pass band filter of 10 Hz and a low‐pass band filter of 500 Hz were applied to remove noise from the signal.

### 2.4 Video collection

Video was collected continuously during each of the treatment periods for subsequent behavior analysis. A GoPro™ camera (Hero7, GoPro Inc., San Mateo, CA) was mounted in a location offering an unobstructed view of the testing area and video collected at a sampling rate of 30 frames per second. A second view of the testing area was recorded at a sampling rate of 30 frames per second (Dell, Round Rock, TX).

### 2.5 Blood sampling

Prior to the start of the study, the hair at the catheter insertion site was clipped to a length of 1.5 mm. On the day of testing, the catheter insertion site was scrubbed with a povidone‐iodine solution, followed by a 70% alcohol solution, and allowed to air dry. Between 1030 and 1100, a jugular catheter (Angiocath, 14 gauge, Becton Dickinson, Inc. Parsippany, NJ, USA) was inserted percutaneously into the left jugular vein using sterile techniques and local lidocaine anesthetic. Horses were allowed to equilibrate for at least 20 min following catheter placement. Blood samples (approximately 14 ml at each collection time point) were collected 15 min prior to the start of the first testing period, at the start of and conclusion of each of the four 3‐min treatment periods, and 30 min after the conclusion of the last treatment period for a total of 10 samples per horse. Samples were placed in pre‐chilled heparin tubes (Vacutainer, Franklin Lakes, NJ, USA) and immediately placed on ice.

### 2.6 sEMG Data Processing

Post processing of data occurred in myoMUSCLE™ (Noraxon, Scottsdale, AZ, USA). EMG signals were filtered using a band‐pass filter (Butterworth, 40 and 450 Hz cutoffs) following current recommendations (St. George et al., 2018). Peak amplitude over a 100 ms duration during social isolation was determined and used for normalization. EMG signals were rectified and then normalized to the peak value obtained previously. The frequency content of the EMG signal was determined using a Fast Fourier transformation (FFT) (MyoMuscle™, Noraxon, Scottsdale AZ, USA). The average rectified value (ARV, %) and median frequency (MF, Hz) were calculated (MyoMuscle™, Noraxon, Scottsdale AZ, USA) for the first 30 s (0–30 s), middle 30 s (75–150 s), and final 30 s (150–180 s) of each treatment period.

### 2.7 Behavior

An ethogram of stress‐related behaviors (Table 1) was developed using previously published ethograms (McDonnell, 2003; McGreevy & McLean, 2010). Three undergraduate students were trained to identify these stress‐related behaviors using short video clips. The training was undertaken by the researcher (EMR) who had extensive previous experience in using the ethogram and training students in using it. Students were required to obtain greater than an 80% level of agreement with the individual providing the training before proceeding. Inter‐rater agreement was assessed within BORIS using Cohen's kappa (Friard & Gamba, 2016). Videos collected during the study were imported into BORIS, a free, open‐source software for video coding (Friard & Gamba, 2016). Each of the three students watched and coded each of the videos for behaviors of interest. Averages of the frequency of observed behaviors from the three observers were used for further analysis.

**TABLE 1:** Stress‐related behavior ethogram developed from McDonnell (2003) and McGreevy and McLean (2010)

Activity	Operational definition
Licking/Chewing	Manipulating mouth and/or tongue, but not ingesting food
Yawning	Deep, long inhalation with mouth widely opened, with jaws either directly opposed or moved side to side
Mouthing	Manipulate object with open mouth. May close teeth on object. Typically seen as horse investigates stimulus
Wood Chewing	Chewing and/or ingesting wooden objects such as stall walls
Tongue Displacement	Tongue hanging far out of the mouth, usually to the side where it dangles loosely
Tongue Lolly	Extraneous movement of the tongue in and out of the mouth
Blowing/Snorting	Forceful expulsion of air from the horse's nostrils
Sniffing	The horse inhales air. Typically seen as the horse investigates the stimulus.
Flehmen	The horse elevates its head and extends its neck with the eyes rolled back, ears rotated to the side, and upper lip everted exposing the upper incisors and adjacent gums
Vocalization	The horse produces a sound of high amplitude and variable frequency.
Biting/nipping*	Opening and rapid closing of the jaws with the teeth grasping the handler, wood, or other objects
Bite Threat*	Opening and rapid closing of the jaws without the teeth contacting the handler, wood, or other objects. The horse's ears are pinned
Ears pinned*	Ears pressed caudally against the horse's head and neck.
Ear Flicking	One or both ears move rapidly forward and backward. Count individual instances
Shake*	The horse rapidly rotates its head, neck, and upper body along the long axis while standing with feet firmly planted
Head Shake	The horse rapidly rotates its head and/or neck along the long axis
Head Tossing	The horse rapidly flicks head forward and up and back down
Stereotypic Head Shaking, Bobbing, Tossing, or Nodding	Repeated, rhythmic head movements
Stomping	The horse strikes the ground with a foot
Pawing	The horse moves its hoof forward and brings it back toward its body by scraping the toe along the ground or just above the ground. Count individual instances
Kick	One or both hind legs lift off the ground and rapidly extend backward toward the handler or other stimulus or object
Kick threat	The hind leg(s) lifts slightly off the ground and under the body in tense “readiness”. May be followed by backward extension of the leg(s)
Rear	The horse raises its forelegs into the air and supports its body in the hind limbs
Tail Swish	The tail moves rapidly in a back and forth or wringing motion. Count individual instances
Defecation	The horse expels feces
Urination	The horse expels urine
Freezing	The horse becomes suddenly immobile and focuses its gaze and orients its ears toward a stimulus
Shying	The horse performs a sudden sideways leap or veers to avoid novel or fear‐provoking stimulus
Spinning*	The horse suddenly changes direction
*Baulk*	The horse stops forward or backward motion before the handler cues it to do so and resists or ignores cues to resume movement
Rushing*	The horse not responding to the cues to slow
*Pulling*	The horse is resistant to pressure applied to the lead and pulls against the lead and shows no deacceleration
Barging*	The horse moves sideways toward the handler. May result in the handler changing course or being displaced by the horse

Note

Behaviors marked with an asterisk were absent in all horses across all conditions. Italicized behaviors could only occur as a result of human–horse interactions and thus, were only assessed during the sham clipping (CLIP) and novel object (NOV) conditions.

### 2.8 Blood sample processing

Hematocrit was measured using the microhematocrit method. Blood samples were then centrifuged at 3000 × g for 12 min. Plasma total protein concentration was measured via refractometry (Leica, Inc., Buffalo, NY, USA). Plasma for the measurement of cortisol was then frozen at −80°C until analyzed. Cortisol concentrations were measured using a solid‐phase chemiluminescent immunoassay system (Immulite, Diagnostic Products Corporation, Los Angeles, CA) previously validated for use in horses (Reimers et al., 1996). Intra‐assay precision was determined by testing a pooled sample 8 times consecutively within the same run. The mean CV was 5.8% for the sample with a mean concentration of 2.16 µg/dl. The interassay precision for a pooled sample (2.15 µg/dl) tested over 7 days over an 8‐day period was 5.5%. Analytical sensitivity was 0.2 µg/dl.

### 2.9 Statistical analysis

Data were inspected for normality. ARV and MF data violated the assumptions of normality. The Box‐Cox procedure was used to determine the appropriate data transformation. ARV and MF data were log transformed prior to further analysis. ARV and MF were analyzed using a mixed model, repeated measures ANOVA with fixed effects of treatment, time point, and period; random effects of horse and side; and interactions of treatment by time point, treatment by side, and time point by side (SAS 9.4, Cary, NC, USA). Insufficient numbers of specific stress‐related behaviors were observed to make analysis by specific behavior valid. The frequency of specific behaviors was summed to acquire the total number of stress‐related behaviors for each horse within each treatment. The sum of stress‐related behaviors was used in further analyses. Behavior data were analyzed using a negative binomial distribution mixed model ANOVA with fixed effects of treatment and period and a random effect of horse (SAS 9.4, Cary, NC, USA). The most appropriate distribution for the model was selected based on visual inspection of the data and comparison of the Akaike Information Criterion (AIC) across models. Inter‐rater agreement was calculated using an intra‐class correlation with two‐way random effects, absolute agreement, and single rater effects. Cortisol concentrations, hematocrit, and plasma total protein were analyzed using a mixed model, repeated measures ANOVA with fixed effects of treatment, time point, treatment by time point interaction, and period and a random effect of horse (SAS 9.4, Cary, NC, USA). Statistically different means were separated using Tukey's method. Significance was set at *p* < 0.05.

## 3 RESULTS

### 3.1 sEMG

#### 3.1.1 Masseter

Log ARV in the *masseter* was significantly affected by treatment (*p* < 0.0001) and time point (*p* = 0.0073), but not side (*p* = 0.727). ISO (0.2 + 1.3%, mean + SD) and CLIP (1.5 + 1.5%) elicited greater (*p* < 0.001) *masseter* log ARV than CON (−1.2 +1.4%). The *masseter* log ARV was greater (*p* = 0.0049) in the initial 30 s (0.2 +1.6%) when compared to the final 30 s (−0.3 + 1.7%). The interaction of treatment and time (*p* = 0.4624) and treatment and side (*p* = 0.9715) had no effect on log ARV in the *masseter*, whereas the time point by side interaction did have an effect (*p* = 0.0205). Log ARV in the left *masseter* during the initial 30 s (0.3 + 1.8%) was greater (*p* = 0.0158 & 0.0018) than log ARV observed during the middle (−0.6 + 1.6%) or final (−0.6 + 1.7%) 30 s in the same muscle (Table 2).

**TABLE 2:** Log average rectified value (ARV) and median frequency (MF) in the left and right *masseter* during the initial (0–30 s), middle (75–105 s), and final (150–180 s) 30 s of the 3‐min control (CON), social isolation (ISO), novel object (NOV), and sham clipping (CLIP) treatments (*n* = 8, 4 males, 4 females)

	*Masseter*			
	Log ARV (%)		Log MF (Hz)	
	Mean	SD	Mean	SD
Left				
CON				
Initial	0.96	1.25	57.3	45.2
Middle	0.66	1.15	65.36	62.52
Final	0.37	0.4	32.27	22.55
ISO				
Initial	3.93	4.53	39.77	33.7
Middle	1.68	2.36	21.81	8.71
Final	1.31	1.04	19.56	4.74
NOV				
Initial	0.72	0.6	20.72	6.6
Middle	0.54	0.58	35.13	28.11
Final	0.46	0.56	49.47	40.54
CLIP				
Initial	16.26	15.6	59.99	0.03
Middle	5.51	6.47	59.96	0.08
Final	6.79	7.79	59.47	14.65
Right				
CON				
Initial	1.59	2.85	46.94	34.86
Middle	0.87	1.24	38.93	34.24
Final	0.58	0.86	51.82	52.66
ISO				
Initial	4.27	4.13	23.53	6.67
Middle	2.34	2.55	22.16	5.38
Final	1.5	0.79	21.69	4.84
NOV				
Initial	0.89	0.71	21.91	5.06
Middle	0.83	0.75	28.75	14.91
Final	0.57	0.55	55.34	51.08
CLIP				
Initial	3.41	3.5	52.84	14.63
Middle	14.56	16.17	59.98	0.03
Final	26.92	59.35	60.37	1.12

Note

ARV values are expressed as a percentage of the peak amplitude observed during social isolation. Data were analyzed with a repeated measures, mixed model ANOVA. Main effects of treatment (*p* < 0.0001) were observed in the log ARV and MF data. A main effect of time (*p* = 0.0073) was observed in the log ARV data.

There was a significant effect of treatment (*p* < 0.0001), but not time (*p* = 0.8167) or side (*p* = 0.5842) on log MF in the *masseter*. ISO (3.1 + 0.4 Hz) and NOV (3.3 + 0.6 Hz) resulted in lower log MF (*p* < 0.0001 & *p* = 0.0427) in the *masseter*, whereas CLIP (4.1 + 0.2 Hz) resulted in higher log MF (*p* < 0.0001) than CON (3.6 + 0.8 Hz). The time by side (*p* = 0.2582) and treatment by side (*p* = 0.9250) interactions had no effect on log MF in the *masseter*. The treatment by time interaction (*p* = 0.0065) influenced log MF in the *masseter*. The middle 30 s of ISO (3.0 + 0.3 Hz) resulted in lower (*p* = 0.0064) log MF than the initial 30 s of CON (3.7 + 0.7 Hz) (Table 2). The final 30 s of ISO (3.0 + 0.2 Hz) resulted in lower (*p* = 0.0283) MF than the initial (3.7 + 0.7 Hz) or middle (3.6 + 0.9 Hz) 30 s of CON. Log MF in the initial 30 s of NOV (3.0 + 0.2 Hz) was lower (*p* = 0.0187 & 0.0069) than log MF in the final 30 s of NOV (3.7 + 0.8 Hz) and the initial 30 s of CON (3.7 + 0.7 Hz). The middle (4.1 + 0.0 Hz) and final (4.1 + 0.2 Hz) 30 s of CLIP resulted in greater (*p* = 0.0218; 0.0027; 0.0287; & 0.0037) log MF than either the middle (3.6 + 0.9 Hz) or final 30 s of CON (3.4 + 0.7 Hz). The initial 30 s of CLIP (3.7 + 0.7 Hz) also resulted in greater (*p* = 0.0142) log MF than the final 30 s of CON (3.4 + 0.7 Hz).

#### 3.1.2 Brachiocephalas

Log ARV in the *brachiocephalas* was significantly affected by treatment (*p* = 0.0007), but not time (*p* = 0.1314) or side (*p* = 0.5943). CLIP (2.2 + 2.0%) elicited greater (*p* = 0.0005) log ARV than CON (0.1 + 1.5%). The interaction of treatment by time (*p* = 0.1864) and treatment by side (*p* = 0.7084) had no effect on log ARV in the *brachiocephalas*, whereas the time by side interaction was significant (*p* = 0.0065). In the left *brachiocephalas*, log ARV during the initial 30 s period (1.2 + 1.7%) was greater (*p* = 0.0041) than log ARV during the final 30 s (0.8 1.7%) (Table 3).

**TABLE 3:** Log average rectified value (ARV) and median frequency (MF) in the left and right *brachiocephalas* during the initial (0–30 s), middle (75–105 s), and final (150–180 s) 30 s of the 3‐min control (CON), social isolation (ISO), novel object (NOV), and sham clipping (CLIP) treatments (*n* = 8, 4 males, 4 females)

	*Brachiocephalas*			
	Log ARV (%)		Log MF (Hz)	
	Mean	SD	Mean	SD
Left				
CON				
Initial	2.84	3.16	69.73	47.83
Middle	3.48	4.38	89.52	56.66
Final	2.63	3.19	70.25	44.53
ISO				
Initial	4.89	4.34	55.73	51.06
Middle	2.88	2.62	38.4	29.34
Final	3.08	3.02	33.36	28.39
NOV				
Initial	4.01	4.52	34.57	24.55
Middle	3.44	5.54	62.45	47.61
Final	3.12	5.33	64.37	55.92
CLIP				
Initial	58.53	92.28	70.53	34.01
Middle	64.63	151.56	75.05	42.58
Final	65.22	158.16	74.35	53.49
Right				
CON				
Initial	2.57	3.37	54.98	22.93
Middle	2.27	2.87	49.96	23.11
Final	2.13	2.56	49.01	24.78
ISO				
Initial	4.65	4.03	41.75	17.09
Middle	3.68	2.61	31.89	11.64
Final	4.05	3.72	35.17	21.79
NOV				
Initial	8.13	11.29	34.41	21.98
Middle	4.43	4.12	53.13	23.02
Final	5.05	5.35	60.52	30.58
CLIP				
Initial	9.18	10.72	57.62	5.48
Middle	47.34	81.13	58.6	3.92
Final	35.23	57.73	59.41	1.6

Note

ARV values are expressed as a percentage of the peak amplitude observed during social isolation. Data were analyzed with a repeated measures, mixed model ANOVA. A main effect of treatment (*p* = 0.0007; 0.0011) was observed in the log ARV and MF data.

Treatment (*p* = 0.0011) had a significant effect on log MF in the *brachiocephalas*, whereas time (*p* = 0.5082) and side (*p* = 0.3391) did not. ISO (3.5 + 0.6 Hz) elicited lower (*p* = 0.0015) log MF than CON (4.0 + 0.6 Hz). The time by side (*p* = 0.6660) and treatment by side (*p* = 0.5584) interactions were non‐significant. The treatment by time interaction (*p* = 0.0010) had a significant effect on log MF in the *brachiocephalas*. Greater log MF (*p* = 0.0230) was recorded in the middle 30 s of CON (4.0 + 0.7 Hz) than the middle 30 s of ISO (3.4 + 0.6 Hz) (Table 3). Similarly, greater log MF (*p* = 0.0442 & 0.0084) was recorded in the *brachiocephalas* during the initial (3.4 + 0.6 Hz) and middle 30 s (4.0 + 0.7 Hz) of CON than the final 30 s of ISO (3.3 + 0.6 Hz). Log MF in the *brachiocephalas* increased (*p* = 0.0027) during NOV from 3.3 log Hz (SD 0.6) during the initial 30 s to 3.9 log Hz (SD 0.6) during the final 30 s. Log MF during the initial 30 s of NOV (3.3 + 0.64 Hz) was also lower (*p* = 0.0131) than log MF during the middle 30 s of CON (4.0 + 0.7 Hz).

#### 3.1.3 Cervical trapezius

Log ARV in the *cervical trapezius* was affected by treatment (*p* < 0.0001), but not by time (*p* = 0.1351), or side (*p* = 0.0771), or the interactions of treatment by time (*p* = 0.1245), time by side (*p* = 0.8625), and treatment by side (*p* = 0.9522). Log ARV in the *cervical trapezius* was lower (*p* < 0.0001 & =0.0258) during CON (−1.0 + 1.7%) then ISO (0.6 + 1.3%) or NOV (−0.2 + 1.3%] (Table 4).

**TABLE 4:** Log average rectified value (ARV) and median frequency (MF) in the left and right *cervical trapezius* during the initial (0–30 s), middle (75–105 s), and final (150–180 s) 30 s of the 3‐min control (CON), social isolation (ISO), novel object (NOV), and sham clipping (CLIP) treatments (*n* = 8, 4 males, 4 females)

	*Cervical trapezius*			
	Log ARV (%)		Log MF (Hz)	
	Mean	SD	Mean	SD
Left				
CON				
Initial	1.58	3.53	40.18	36.32
Middle	1.95	4.86	56.8	50.1
Final	1.37	3.24	46.2	52.28
ISO				
Initial	4.95	7.42	25.46	9.94
Middle	2.01	2.02	18.35	3.11
Final	2.79	2.82	19.37	3.69
NOV				
Initial	1.31	0.98	45.1	54.15
Middle	1.18	1.27	37.54	52.35
Final	1.18	2.11	54.5	52.47
CLIP				
Initial	1.02	0.93	54.26	44.58
Middle	0.83	0.93	53.94	43.82
Final	0.78	1.3	64.78	38.97
Right				
CON				
Initial	2.62	5.86	58.55	53.58
Middle	2.84	6.62	56.09	50.97
Final	2.83	6.69	47.78	51.91
ISO				
Initial	6.84	9.85	24.82	7.16
Middle	2.75	3.03	21.59	8.06
Final	3.61	2.17	28.89	20.75
NOV				
Initial	2.57	3.51	20.15	2.84
Middle	5.04	10.52	20.69	3.83
Final	1.84	2.6	54.62	49.21
CLIP				
Initial	2.67	5.79	24.13	15.03
Middle	3.92	9.04	49.59	19.31
Final	3.43	7.31	55.99	11.3

Note

ARV values are expressed as a percentage of the peak amplitude observed during social isolation. Data were analyzed with a repeated measures, mixed model ANOVA. A main effect of treatment (*p* < 0.0001; =0.0038) was observed in the log ARV and MF data. A main effect of time (*p* = 0.0007) was observed in the MF data.

Significant effects of treatment (*p* = 0.0038) and time (*p* = 0.0007) were observed in log MF of the *cervical trapezius*, but no effect of side (*p* = 0.8558) was observed. Log MF during CON (3.6 + 0.8 Hz) was greater (*p* = 0.0106) than log MF during ISO (3.1 + 0.3 Hz) in the *cervical trapezius*. Log MF during the final 30 s (3.6 + 0.7 Hz) was greater (*p* = 0.0026 & 0.0283) than the initial (3.3 + 0.7 Hz) and middle (3.4 + 0.7 Hz) 30 s. The interactions of time by side (*p* = 0.1803) and treatment by side (*p* = 0.6169) were non‐significant, whereas the effect of treatment by time (*p* < 0.0001) had a significant effect on log MF in the *cervical trapezius*. The middle 30 s of ISO (3.0 + 0.2 Hz) elicited lower (*p* = 0.0144) log MF than the middle 30 s of CON (3.7 + 0.8 Hz) (Table 4). The final 30 s of NOV (3.7 + 0.8 Hz) elicited greater (*p* = 0.0145 & 0.0025) log MF than the initial (3.2 + 0.7 Hz) or middle (3.1 + 0.7 Hz) 30 s of NOV. Log MF in the *cervical trapezius* increased (*p* = 0.0006) from the initial 30 s (3.4 + 0.7 Hz) to the final 30 s (4.0 + 0.5 Hz) during CLIP.

#### 3.1.4 Longissimus dorsi

Log ARV in the *longissimus dorsi* was unaffected by treatment (*p* = 0.1271), time (*p* = 0.4254), side (*p* = 0.5630), and the interactions of treatment by time (*p* = 0.0778), time by side (*p* = 0.6933), and treatment by side (*p* = 0.9091) (Table 5).

**TABLE 5:** Log average rectified value (ARV) and median frequency (MF) in the left and right *longissimus dorsi* during the initial (0–30 s), middle (75–105 s), and final (150–180 s) 30 s of the 3‐min control (CON), social isolation (ISO), novel object (NOV), and sham clipping (CLIP) treatments (*n* = 8, 4 males, 4 females)

	*Longissimus dorsi*			
	Log ARV (%)		Log MF (Hz)	
	Mean	SD	Mean	SD
Left				
CON				
Initial	7.59	10.56	81.98	61.55
Middle	7.88	8.94	84.18	61.14
Final	7.47	9.51	66.05	53.18
ISO				
Initial	11.33	13.73	57.56	53.98
Middle	10.55	15.95	43.08	32.94
Final	11.71	15.54	35.21	20.68
NOV				
Initial	18.41	28.05	44.96	20.84
Middle	16.51	26.54	85.63	61.1
Final	18.23	29.62	82.11	64.52
CLIP				
Initial	20.3	31.2	92.27	74.17
Middle	19.22	27.88	113.87	67.89
Final	18.43	27.13	120.51	74.75
Right				
CON				
Initial	6.09	8.37	84.45	68.41
Middle	6.45	8.44	113.88	72.08
Final	6.18	8.41	88.82	62.93
ISO				
Initial	8.66	10.9	42.97	33.5
Middle	7.77	12.82	47.69	35.8
Final	8.32	11.43	45.76	50.6
NOV				
Initial	10.52	11.39	35.65	20.61
Middle	10.1	8.96	67.38	44.71
Final	9.96	9.06	100.6	48.55
CLIP				
Initial	9.67	9.99	139.4	56.95
Middle	10.22	9.45	102.91	49.96
Final	9.15	8.03	132.05	58.9

Note

ARV values are expressed as a percentage of the peak amplitude observed during social isolation. Data were analyzed with a repeated measures, mixed model ANOVA. A main effect of treatment (*p* = 0.0001) was observed in the log MF data.

Log MF in the *longissimus dorsi* was affected by treatment (*p* = 0.0001), whereas time (*p* = 0.1865) and side (*p* = 0.3812) had no effect. Log MF was greater (*p* = 0.0068) during CON (4.1 + 0.9 Hz) than during ISO (3.5 + 0.7 Hz). The time by side (*p* = 0.3989) and treatment by side (*p* = 0.7435) interactions had no effect on log MF in the *longissimus dorsi*, whereas the treatment by time interaction (*p* = 0.0385) had a significant effect. The final 30 s of ISO (3.5 + 0.6 Hz) elicited lower (*p* = 0.0417) log MF than the middle 30 s of CON (4.2 + 0.9 Hz) (Table 5). Log MF in the *longissimus dorsi* increased (*p* = 0.0098) from the initial 30 s (3.6 + 0.6 Hz) to the final 30 s (4.3 + 0.7 Hz) during NOV.

### 3.2 Behavior

The total number of stress‐related behaviors was greater (*p* = 0.0019) during ISO (25 + 27) than CON (6 + 9). These values also represent the range of the total number of stress‐related behaviors observed (Table 6). Raters obtained a high degree of agreement with an intra‐class correlation of 0.89.

**TABLE 6:** Total number of stress‐related behaviors displayed during control (CON), social isolation (ISO), novel object (NOV), and sham clipping (CLIP) (*n* = 8 horses; 4 mares, 4 geldings)

	CON	ISO	NOV	CLIP
Stress‐related behaviors [mean (SD)]	6 (9)	25 (27)^a^	11 (11)	7 (5)

Note

Data were analyzed with a negative binomial distribution mixed model ANOVA with a Tukey's *post hoc* adjustment and significance set at *p* < 0.05.

a Differs significantly from CON (*p* = 0.0019).

### 3.3 Hematocrit

There was a main effect of time point on hematocrit (*p* = 0.0013) with samples taken at the end of the 3‐min periods being greater (34 + 4%) than samples taken at the start of the 3‐min periods (32 + 3%). There was no main effect of treatment on hematocrit (*p* = 0.4300). The interaction between treatment and time point was significant (*p* = 0.0112). During ISO, hematocrit increased (*p* = 0.0009) from 32% (SD 3) at the start of the 3‐min period to 37% (SD 4) at the end of the 3‐min period (Table 7). Hematocrits ranged from 31% (SD 2) at the beginning of the CLIP period to 37% (SD 4) at the conclusion of the ISO period (Table 7).

**TABLE 7:** Hematocrit (%) at the beginning (pre) and end (post) of control (CON), social isolation (ISO), novel object (NOV), and sham clipping (CLIP) periods (*n* = 8 horses; 4 mares, 4 geldings)

	Hematocrit (%)		
	Mean	SD	*p*‐value
CON			
Pre	32	5	
Post	32	3	0.7368
ISO			
Pre	32	3	
Post	37	4	**0.0009**
NOV			
Pre	32	3	
Post	32	5	0.3257
CLIP			
Pre	31	2	
Post	33	4	0.8325

Note

Data were analyzed with a repeated measures, mixed model ANOVA with a Tukey's *post hoc* adjustment and significance set at *p* < 0.05. *p* values represent comparisons between pre‐ and post‐measurements within a treatment. Hematocrit (%) was elevated in the post‐measurement following ISO as compared to the pre‐measurement. The bolded value indicates a significant (*p* < 0.05) for the comparison between pre‐ and post‐time points within a treatment.

### 3.4 Plasma total protein

There was no effect of treatment (*p* = 0.6078), time point (*p* = 0.9162), or treatment by time point interaction (*p* = 0.7718) on plasma total protein. Plasma total protein ranged from 6.18 g/dL (SD 0.53) at the end of the CLIP period to 6.53 g/dL (SD 0.27) at the end of the ISO period (Table 8).

**TABLE 8:** Plasma total protein (g/dl) at the beginning (pre) and end (post) of control (CON), social isolation (ISO), novel object (NOV), and sham clipping (CLIP) periods (*n* = 8 horses; 4 mares, 4 geldings)

Plasma total protein (g/dl)		
	Mean	SD
CON		
Pre	6.40	0.27
Post	6.38	0.32
ISO		
Pre	6.36	0.24
Post	6.53	0.29
NOV		
Pre	6.40	0.29
Post	6.39	0.20
CLIP		
Pre	6.33	0.25
Post	6.18	0.53

Note

Data were analyzed with a repeated measures, mixed model ANOVA with a Tukey's *post hoc* adjustment and significance set at *p* < 0.05. No significant differences are present.

### 3.5 Cortisol

Treatment (*p* = 0.6665), time point (*p* = 0.5477), and the treatment by time interaction (*p* = 0.2280) had no effect on plasma cortisol concentrations. Cortisol concentrations ranged from 2.75 µg/dl (SD 0.93) at the end of the CLIP period to 3.04 µg/dl (SD 1.19) at the end of the ISO period (Table 9).

**TABLE 9:** Cortisol concentration (µg/dl) at the beginning (pre) and end (post) of control (CON), social isolation (ISO), novel object (NOV), and sham clipping (CLIP) periods (*n* = 8 horses; 4 mares, 4 geldings)

Cortisol (µg/dl)		
	Mean	SD
CON		
Pre	2.91	0.77
Post	3.01	0.71
ISO		
Pre	2.86	1.02
Post	3.04	1.19
NOV		
PRE	3.00	0.74
post	2.93	0.55
CLIP		
Pre	2.82	0.86
Post	2.75	0.93

Note

Data were analyzed with a repeated measures, mixed model ANOVA with a Tukey's *post hoc* adjustment and significance set at *p* < 0.05. No significant differences are present.

## 4 DISCUSSION

The hypothesis was partially supported by the results as significant alterations in muscle activity, behavior, and hematocrit were observed under conditions of acute stress, but no change in plasma cortisol was observed.

The higher ARV observed in the *masseter* during sham clipping and social isolation as well as the *brachiocephalas* during sham clipping indicate greater muscle activity in these muscles. Greater ARV and other measures of EMG signal amplitude are associated with greater force production during isometric contractions by the muscle, thus making such measures commonly used estimates of muscle force production (Cheung et al., 1998; Lawrence & De Luca, 1983). The relationship between signal amplitude and force generation is not always as straightforward as this as it is influenced by a variety of factors (DeLuca, 1997). Despite these limitations, the interpretation of greater ARV in the *masseter* during sham clipping and social isolation and the *brachiocephalas* during sham clipping as muscular tension or increased muscle activity seems warranted and similar to conclusions reached by other researchers when interpreting measures of EMG amplitude obtained during dynamic movements (Crook et al., 2010; Kienapfel, 2015; Robert et al., 2010; Zsoldos et al., 2010) The preponderance of literature pertaining to EMG in horses has been undertaken during locomotion, which elicits primarily anisometric, dynamic contractions rather than the isometric, static contractions needed for the most robust interpretation of results (Valentin & Zsoldos, 2016; Williams, 2018). The current study suffers from the same limitation and this should be borne in mind when interpreting the EMG results obtained. Horses engaged in little locomotory activity during the sham clipping and novel object exposure making it unlikely that the changes in ARV in the *masseter* and *brachiocephalas* stem from increased locomotion or increased movement in the area of interest. The increase in ARV in the *cervical trapezius* during social isolation and novel object exposure must be interpreted with greater care as horses engaged in more locomotion during these conditions, based on the behavioral measures and observation of the recorded video footage. The *cervical trapezius* is active during locomotion in the horse (Robert et al., 1998). The higher ARV in the *cervical trapezius* observed during social isolation and novel object exposure may reflect the increased locomotion rather than muscular tension. The absence of changes in ARV in the *longissimus dorsi* is surprising given the changes found by Lesimple et al. (2012) in the resting amplitude of spinal muscles in different populations of horses. The changes observed in the study were taken to reflect the welfare status of the horse and as such may represent consequences of chronic rather than acute stress (Lesimple et al., 2012). The acute stress (3‐min duration) used in the current study may have been insufficient to elicit changes in the muscular activity and tension in the *longissimus dorsi*. Postural affects could also come into play as horses in the current study were free to move the position of the head and neck, thus, also allowing freedom of movement through the spine and back. Further exploration of the differences in muscular responses elicited by acute and chronic stress could be of interest.

The untransformed ARV values recorded in this study were less than ten percent in most conditions. These values are lower than those typically reported in the literature (St. George et al., 2018). These studies, however, report values during locomotion which are expected to be higher as greater muscle activation would be needed during locomotion than rest. The data collected in the current study also had a high level of variability despite adherence to recommended best practices in data processing which has been shown to decrease variability and improve sensitivity of analysis (St. George et al., 2019). High variance within and between subjects is common in EMG data (Williams, 2018). The recommendation of testing within subjects and across a single day for each horse was adhered to in the current study (Williams, 2018). Removal of all hair from the EMG electrode attachment was impossible; however, hair was clipped as short as possible, and attachment sites were scrubbed before electrode attachment. Further steps (repeated scrubbing or further hair removal) were taken prior to data collection if an unacceptable level of impedance was observed making it unlikely the electrode preparation and attachment imposed limitations in the current study. Another of these recommendations for best practice is to normalize the recorded amplitudes to the maximum value for that individual as raw amplitude can be highly variable and is influenced by the muscle under consideration, the individual, and the electrode placement (St. George et al., 2019; Valentin & Zsoldos, 2016). The gold standard for normalization is a maximal voluntary contraction, where maximum contraction is voluntarily sustained over a period of time. Maximal voluntary contraction is impossible to achieve in the horse, so the recommendation is normalization to the peak contraction obtained during the movement (Valentin & Zsoldos, 2016). Conditions in the current study made determination of a normalization standard difficult as there was not one movement or locomotory pattern under study. If this method of assessing stress is used in the future, we would recommend choosing a set of movements or behaviors likely to elicit muscular contraction in the muscles of interest for use in normalizing collected data. Such an approach would be likely to reduce variability in the data.

Social isolation consistently resulted in lower MF across all four of the muscles of interest. The most frequent interpretation of lowered MF is as an indicator of muscular fatigue (Colborne et al., 2001; Williams, 2018). While this interpretation is most correctly applied during isometric muscle contraction, it has also been applied to dynamic movement patterns, especially in the horse (Cheung et al., 1998; Colborne et al., 2001; Williams et al., 2013). Fatigue as an explanation for the lower MF observed during social isolation is possible given the high level of activity observed, but may not be of physiological significance and is unlikely given the short duration of the stressor. This interpretation could also be applied to the lower MF recorded in the *masseter* during novel object exposure, but the same concerns as noted above are present. The increase in MF recorded in the *masseter* during sham clipping does not fit this pattern. Increases in signal frequency have been observed with increased force production (Doheny et al., 2008). Thus, a greater force may have been produced by the *masseter* during sham clipping as evidenced by increased ARV and MF. The pattern of muscle activation may also play a role in the differing responses seen in ARV and MF over the four conditions and four muscles as visual inspection of the EMG trace revealed sustained muscle activation in the *masseter* during sham clipping and internment activation in other conditions and muscles. Similarly to the untransformed ARV, untransformed MF values were often lower than those reported in the literature (Colborne et al., 2001; Williams et al., 2013). The upper range reported in the current study is similar to values reported in the literature and thus, lower values are likely reflective of the differing context since locomotion was not the primary focus of this study (Colborne et al., 2001). Research has also suggested that muscle fiber type and size can affect median frequency indicating comparison of values from different muscles should be undertaken with caution (Kupa et al., 1995). MF results should also be interpreted with great caution in the present study given the lack of standardization in locomotion across the testing conditions.

The statistically significant increase in stress‐related behaviors during social isolation was expected as similar responses have been reported in other studies (Lansade et al., 2008a). The lack of a statistically significant increase in stress‐related behaviors during novel object exposure and sham clipping was unexpected. This result can be attributed in part to the presence of a human handler in these two conditions as this could be construed to restrict behavioral stress responses in the horse. If this study was repeated or a similar study was undertaken, behavioral expression and locomotory activity should be restricted equally across all conditions. The presence of a handler and conspecifics should also be considered and kept as consistent as possible across testing contexts or accounted for in data analysis. Variability in the number of stress‐related behaviors displayed was high across all conditions. This variability is unsurprising given that personality traits such as anxiousness, sociability, and aggressiveness are associated with behavioral reactions during stressful situations (Ijichi et al., 2013; Peeters et al., 2012). Studies assessing stress in horses should attempt to control or account for this variation between individuals, especially if behavior is used as an indicator of stress. The use of an array of measures can also help circumvent the issue of variation between individuals.

The lack of a cortisol response to the acute stressors applied in this study likely arises from the short duration of the stress (3 min per stressor) and the sampling timeline used. Cortisol peaks 30 min after a perturbation to the bodily system, such as exercise (McKeever et al., 2014). The values reported in this study are comparable to other values reported for horses at rest during the nadir of cortisol secretion (Johnson & Malinowski, 1986). Cortisol secretion follows a circadian rhythm with concentrations peaking in the early morning (Cordero et al., 2012; Johnson & Malinowski, 1986). The study times used allowed for sampling when cortisol concentrations would be low, and increases were more likely to be detectable (Brandenberger et al., 1982; Dickstein et al., 1991).

Increases in total plasma protein are typically associated with fluid shifts in the body that occur during activities such as exercise (McKeever et al., 1993). The lack of statistically significant changes in total plasma protein indicates that removal of access to water during the study had little effect on fluid balance in the horses. Thus, there is no need to account for possible fluid shifts when considering plasma cortisol concentrations. The values for total plasma protein reported in the present study are well within the ranges reported in previous studies of horses (Stewart et al., 1977).

Hematocrit values reported in this study fall within the lower range of hematocrit values reported in the literature for horses at rest which is likely a reflection of the horses’ low fitness level and high degree of familiarity with the study procedures and facilities (Rose & Allen, 1985). The increase in hematocrit during social isolation is indicative of activation of the SAM axis with the release of catecholamines and subsequent splenic contraction. Mobilization of red blood cells from the spleen occurs within 30 to 60 s of stimulation meaning an increase would be expected to occur within the 3 min time frame used in the study (Persson & Lydin, 1973). While the increase in hematocrit reported in this study (5%) is lower than increases observed during exercise, the change is comparable to the difference reported by Stewart et al. (1977) between excited and placid timid, or apprehensive horses (McGowan & Hodgson, 2014).

Taken together, the results obtained in this study indicate an inconsistent response to potential stressors as social isolation elicited changes in hematocrit and behavior, whereas sham clipping elicited changes in surface EMG measures and novel object exposure elicited very few changes. Even though these conditions were carefully selected based on their use in eliciting stress responses in previous studies, it is possible they were insufficient in the current context to induce the desired response. The lack of changes in behavior, hematocrit, and cortisol during sham clipping and novel object exposure do not rule out the possibility of horses experiencing distress during these procedures. In fact, these results may point toward the need for more ways of measuring and evaluating stress in horses. Changes in muscle activity measured via sEMG in the current study alone are insufficient evidence to conclude a stress response was invoked given the limitations associated with these measures in the current study. With this in mind, we recommend further investigation of muscle tension as an indicator of stress and distress in horses along with other novel measures of stress.

## 5 CONCLUSION

Muscular tension measured via surface EMG is a promising indicator of distress in horses. The inclusion of muscular tension as a measure of stress in future studies can help researchers assemble an array of variables to provide a more nuanced understanding of stress in the horse. This measure may be particularly relevant for horses with a personality or experimental conditions that limit the behavioral expression of stress.

## CONFLICT OF INTEREST

The authors have no conflict of interest to declare.

## AUTHOR CONTRIBUTIONS

EMR, KM, and KHM conceptualized and designed the study. EMR, HCM, KM, and KHM collected samples and data, performed analyses, and interpreted the data. EMR, HCM, KM, and KHM drafted the manuscript or revised it critically for important intellectual content.
